# Science Production in Germany, France, Belgium, and Luxembourg: Comparing the Contributions of Research Universities and Institutes to Science, Technology, Engineering, Mathematics, and Health

**DOI:** 10.1007/s11024-017-9327-z

**Published:** 2017-07-10

**Authors:** Justin J.W. Powell, Jennifer Dusdal

**Affiliations:** Institute of Education and Society, University of Luxembourg, 11, Porte des Sciences, 4366 Esch-sur-Alzette, Luxembourg

**Keywords:** Science production, University, Research institute, Institutionalization, Science Citation Index Expanded, Science, Technology, Engineering, Mathematics, and Health, Belgium, France, Germany, Luxembourg

## Abstract

Charting significant growth in science production over the 20th century in four European Union member states, this neo-institutional analysis describes the development and current state of universities and research institutes that bolster Europe’s position as a key region in global science. On-going internationalization and Europeanization of higher education and science has been accompanied by increasing competition as well as collaboration. Despite the policy goals to foster innovation and further expand research capacity, in cross-national and historical comparison neither the level of R&D investments nor country size accounts completely for the differential growth of scientific productivity. Based on a comprehensive historical database from 1900 to 2010, this analysis uncovers both stable and dynamic patterns of production and productivity in Germany, France, Belgium, and Luxembourg. Measured in peer-reviewed research articles collected in Thomson Reuters’ Science Citation Index Expanded, which includes journals in the fields of Science, Technology, Engineering, Mathematics, and Health, we show the varying contributions of different organizational forms, especially research universities and research institutes. Comparing the institutionalization pathways that created the conditions necessary for continuous and strong growth in scientific productivity in the European center of global science emphasizes that the research university is the key organizational form across countries.

## Institutionalizing and Organizing Science Production in STEM Fields

Charting huge growth in science production over the 20th century in four EU member countries, we analyze the development and contemporary state of universities and research institutes that bolster Europe’s position as a key region in global science. On-going internationalization (and Europeanization) of higher education and science challenges traditional nation-based studies. In response, neo-institutional analyses have explored the powerful diffusion of worldwide ideas and norms in science (Drori et al. 2003). This framework emphasizes global similarities, with higher education expanding worldwide (Meyer 2009) and the “super research university” a powerful contributor to the “schooled society” (Baker 2014). At the same time, despite convergence pressures, comparative institutional analyses show persistent differences in higher education systems (Powell and Dusdal 2016, in press), yet the increasing significance of the research university across countries.

Our sample of four countries reflects the history and development of the research university as well as independent research institutes. Belgium, France, Germany, and Luxembourg, neighboring countries in Western Europe, are founding members of the forebears of the European Union (EU) and in the contemporary Bologna process. They are connected in multilevel governance and participate in myriad joint education and research programs, such as Erasmus. At the intersection of the Germanophone and Francophone worlds, these countries differ in languages and cultures, in demographics and geography, and in resources devoted to education and science, yet not necessarily consistently. In an era of internationalization, massive growth in the scientific output of these four countries simultaneously reflects competition and collaboration. Our comparison uncovers contrasting investments in research and development (R&D) and variable institutionalization of higher education and science systems.

Measured in papers published in leading peer-reviewed journals of the Science Citation Index Expanded (SCIE), the volume of scientific output differs, sometimes unexpectedly, according to institutionalized structures of higher education and research. For example, the relative importance of universities, research institutes, and firms differs across cases, even as the research university’s contribution rises. The overall scientific output in science and technology disciplines increased dramatically over the 20th century, with Europe losing, but regaining its position as the global “center of gravity” (Zhang et al. 2015). Together, these four countries contribute considerably to global science production as their scientists publish a vast number of scientific papers. While all invest in education and science at all levels, as measured in absolute terms and per capita, we find important differences in productivity, especially over the post-WWII period. On the basis of comprehensive, historical data of science, technology, engineering, and mathematics disciplines (STEM) as well as health, we measure the volume of science produced, tracing in particular the development of research universities and institutes as the two major organizational forms that host scientists producing peer-reviewed publications in specialized scientific journals.

The selected countries differ in science policies, higher education and science systems, and internationalization. They also share borders and manifest extensive collaboration and competition since the foundings of the earliest universities. Among all science-producing organizational forms, how much do universities and non-universities, especially extra-university research institutes, proportionately, contribute to scientific productivity? How do these countries, with varying institutionalization of universities and institutes, compare in the production of STEM research?

To address these questions, we proceed as follows: We first discuss regional trends in science production and locate the four countries within the European center of science. Reviewing our historical and quantitative data and methods, we present findings on developing research-producing structures and science production in each country, emphasizing the strong and growing contribution of research universities. Finally, we compare across countries to better understand how systems of higher education and research largely responsible for scientific productivity were institutionalized.

## Science Production in Western Europe

Higher education and research, transmitting and producing knowledge in the *lingua franca* of the day, are thoroughly worldwide activities. Along with changes in the “center” of science—France around 1800, Germany from 1840, and the US since WWI (Ben-David 1984)—the language of science shifted from French to German to English, leading to the current dominance of journals published in English. The case selection portrays the shifting significance of these three official languages. Today, English everywhere provides a (necessary) common communication platform, especially in STEM disciplines examined here.

Analyzing millions of original articles published since 1900 manifests unprecedented growth in the global pursuit of science: Beginning just after mid-century, pure exponential growth builds on contrasting concurrent trends—rising competition between countries and international collaboration across national borders (Zhang et al. 2015). Home to many of the oldest research universities and other organizational forms, such as academies and research institutes, Europe is at the heart of scientific productivity between North America and East Asia (on Russia and China, see Oleksiyenko 2014). Universities and extra-university research institutes provide spaces and support for intercultural collaboration and learning and for scientific discovery, extending massive educational expansion in societies worldwide (Schofer and Meyer 2005), as countries benefit from the strength of research universities (Baker 2014).

Today, all countries invest in R&D and in higher education, the smaller ones often doing so through a single national university (Luxembourg) or a set of strong research universities in different regions (Belgium’s language communities of Flanders and Wallonia). The two larger countries (France, Germany) maintain differentiated systems of universities (of varying size and prestige) and extra-university research institutes (connected in large umbrella associations or coordinated by government agencies). This requires considerable state investment: “The most consequential scientific revolutions of our time could not have happened in universities without massive government and/or corporate support” (Kennedy 2015: 314). Alongside the key indicator for the quantitative measurement of science (publications), institutional, personnel, or financial indicators likewise facilitate estimates of scientific growth and development. Rising science productivity demands commensurate resources (Weingart [2001] 2015), regardless of the actual, difficult-to-measure impact of any individual scientific article. While research on the relationship between R&D funding and demonstrated knowledge production is limited, studies confirm the general positive relationship between research funding and publication output (see Rosenbloom et al. 2014 on chemistry).

**Fig. 1:**
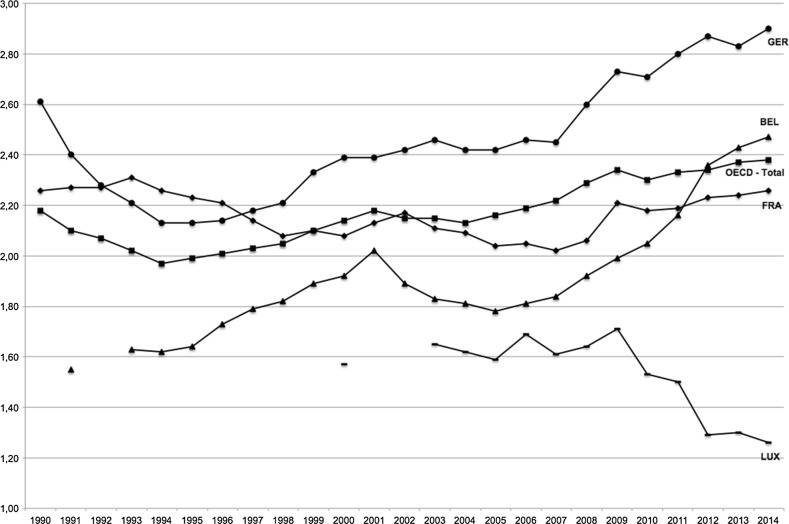
Research Investments in Germany, France, Belgium, and Luxembourg (GERD as a proportion of GDP—“research intensity”), 1990–2014. *Source* OECD.Stat (2017): Main Science and Technology Indicators; last accessed 2017.06.08

Comparing the level of gross domestic expenditures on R&D (GERD) as a proportion of GDP—“research intensity”—in the four countries shows considerable variance. To estimate the impact of investments in science, a time lag should be recognized. Here, we select GERD two years before the last publications gathered in our database were published to indicate the relevant investments (2008). The OECD mean was 2.29% while the EU-15 mean was 1.91%. Germany had increased its R&D investments to 2.60%. France has been relatively stable above 2% since 2000 (2.06% in 2008). Belgium invested 1.92%; just below France, but far lower than Germany. Luxembourg had a mean of 1.64%, lower than its three neighboring countries. None have reached the EU target of 3% to be invested in “innovation.” Thus, these countries’ investments vary by a factor of two (see Fig. 1).

Derived from the aforementioned assumptions, we would expect that Luxembourg has produced considerably less scientific publications per capita than Belgium, France, or Germany, despite its recent increasing investments. Given their larger investments, Germany, Belgium, and France should produce more. Competition amongst the strongest science countries has risen in an age of self-proclaimed excellence and comparative indicators, but collaboration has also grown dramatically across cultures and countries (Powell et al. in press). Our selection of countries reflects higher education and science systems with different institutional structures to examine and compare which institutional setting(s) provide the best conditions for scientific production and productivity.

The institutional settings and organizational forms in which research is conducted affect overall capacity and scientific productivity. Establishing new organizations involves high costs and myriad challenges, especially for small states due to limited native highly qualified human capital and lack of economies of scale (Martin and Bray 2011), but large countries also make challenging choices about which types of higher education and research organizations will be most productive. State investment in science is often divided between universities and extra-university research institutes that have varying emphases (fundamental to applied) and with differing degrees of academic freedom. Research institutes and universities alike struggle to develop their reputations, which often requires generations of scholarship. Yet while research institutes may focus mainly on immediate scientific output, universities must balance research and teaching, the unity of which remains the foundational principle of the modern research university (Ash 1999). Universities receive less than a fifth of all funds: only 18% (Germany), 21% (France), 20% (Belgium), and 19% (Luxembourg) of the overall expenditures in R&D went to the higher education sector; remarkably similar proportions given the contrasting extent of university institutionalization. We might expect universities to produce proportionally less due to their modest proportion of funds and given their other missions of teaching and public service. Indeed, although universities of all kinds experience “academic drift” and scientists are intrinsically-motivated to conduct research, universities in many countries are challenged by the lack of resources as states retrench their commitments to public higher education, even as the costs of tertiary education continuously rise (OECD 2014). Increasing science budgets across Europe have, when calculated as a proportion of GDP, not kept pace with the rhetoric extolling the benefits of science and innovation (OECD 2014). The rationale and vision shared by many governments of how to build capacity for science rests on the notion that infrastructure for research cannot be provided only by industry; the state must invest in the so-called “knowledge triangle”—the beneficial combination of research activity, specialized education/training and innovation that advances knowledge (European Commission 2010: 3). Predictably, however, despite the state investments, higher education and science systems and the resultant scientific productivity vary considerably across countries given long-term institutionalization (and intergenerational exchange) needed to build environments that successfully support scientific discovery.

In Europe as elsewhere, the supranational dimension is becoming more influential, exemplified in intergovernmental processes leading to standardization in higher education (Bologna process) and in such increasingly influential government initiatives (e.g., Horizon 2020, the EU’s framework program for science) and organizations (European Research Council) that fund European science on the frontier (Flink 2016; Hoenig 2017). Focusing on four neighboring countries within the European center of scientific production, we compare growth over time in their higher education and research systems and the resulting scientific productivity. We analyze the institutionalization of their systems—following different models and compositions of organizational forms and fields structured over centuries—and research policies, especially their investments in R&D.

We measure science production on the basis of a dataset from Thomson Reuters (Web of Science) of published papers in selected science and technology disciplines, including health. The increasing role of conference proceedings and other publication formats in STEM disciplines with high growth rates (e.g., computer sciences and engineering) are only partially reflected in the SCIE database. Nevertheless, peer-reviewed journal articles are the most important and traditional type of publications in these fields, next to patents—and the growth rate of scientific publications is still increasing overall, with disciplinary differences (Olesen Larsen and von Ins 2010). By including health-related disciplines, this dataset may inflate the productivity of universities with academic teaching hospitals. Focusing on STEM disciplines, we examine research produced in universities and research institutes that rely heavily on public funds.^1^ Selecting a set of disciplines is necessary, because disciplines form “the primary unit of internal differentiation of the modern system of science” (Stichweh 1992: 4). While official publication figures under-represent the true extent of scientific productivity and SCIE data is biased towards the English language, nevertheless, peer-reviewed research articles indexed in the Web of Science or Elsevier’s Scopus database are the key source for most bibliometric analyses (Glänzel 2016).

## The Institutionalization of Research Universities and Research Institutes

Theoretically, we apply a sociological neo-institutional framework to explore and explain both the tremendous expansion of higher education and science across the world and considerable differences across time and space in the institutional settings, organizational fields and forms, and organizations that produce the most research (see Scott 2015). Science, as a social institution that follows internal social norms and rules (Merton 1942), in turn provides the foundations for the production of scientific knowledge (Weingart [2003] 2013). As communities of organizations, organizational fields reflect the interrelationships of diverse organizations sharing an environment (Aldrich and Ruef 1999). Within a field, particular organizational forms share similar functions and organizations share a common network; this is quite true within scientific communities that, spanning the globe, rely on familiar organizational forms, such as the university. Organizations are defined as social structures established to achieve specific goals through the coalition of actors embedded in an institutional environment (Scott [1995] 2014). The focus on the organizational field and organization levels enables an analysis of differential contributions to scientific productivity.

Universities with their institutional character are assumed to be the most appropriate organizational form for creating significant scientific knowledge, providing the setting for research simultaneously with teaching each new generation of scientists. Alongside universities, diverse state-supported research institutes constitute another pillar of modern science. These various organizational forms undergird local, regional, and national economic development even as they expand human rights and individuals’ capabilities (Meyer 2009). Increasingly, individual well-being and societal futures rely on scientific discoveries, generated more than ever in research universities that remain key contributors of scientific outputs (Baker 2014). Despite numerous hypotheses regarding the transformation of knowledge production (Nowotny et al. 2003), the variable contributions of different organizational forms across decades and in different countries has been rarely addressed in explicit comparisons (but see Dusdal 2017). We begin such exploratory analysis here, focusing on universities and research institutes as the primary organizational forms producing state-funded research. Research universities are characterized by fundamental principles of the nexus of research and teaching, freedom to teach and to study, autonomy and commitment to science as well as the granting of doctoral degrees. Research institutes contribute less to teaching, instead focusing on research, often in well-resourced, cutting-edge facilities.

In comparison, these four countries differ in the scale and scope of their systems—and, as analyzed below—in the distribution and developmental pathways of their universities and research institutes. Universities have contrasting positions, especially due to differences in the institutionalization of higher education and research. While Belgium, France, and Germany have centuries-old, world-renowned research universities, Luxembourg has among the youngest in Europe (Powell 2015). Both Germany and France also have well-established extra-university research institutes, often linked in extensive associations that contribute hugely to these countries’ scientific output—and are world leaders (Oleksiyenko 2014: 498). Especially in Belgium, but also in Germany, research universities are most significant organizations for producing science. In France, and especially in Luxembourg, research institutes have produced most STEM science; however, universities are catching up.

**Fig. 2:**
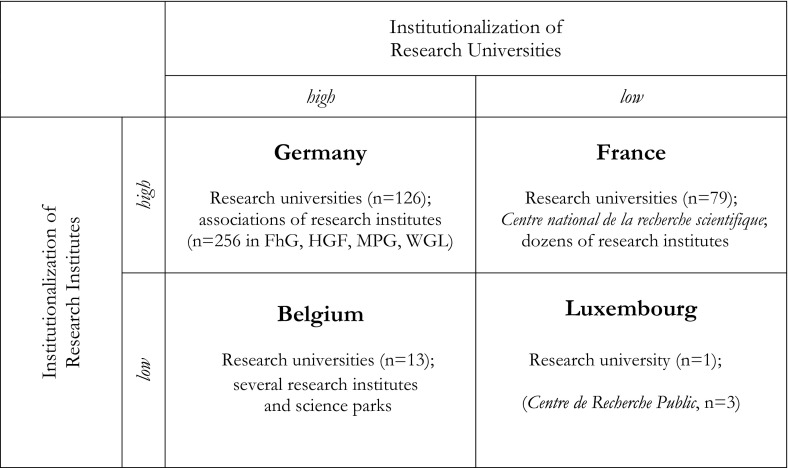
The Institutionalization of Universities and Research Institutes in Germany, France, Belgium, and Luxembourg. *Source* Authors’ representation

According to the volume of produced STEM papers and to historical reach, we sketch the development of universities and research institutes in Germany and France, then in Belgium and Luxembourg, showing how capacity for producing scientific papers has grown over time. To understand the level and extent of institutionalization of universities and research institutes in each country, we display these systems depending on the development of the (non)university sectors (Fig. 2). Europe has among the oldest and leading research universities worldwide, such as the Paris-Sorbonne University (founded 1150), University of Heidelberg (1386) or Catholic University Leuven (1425) that produce large numbers of publications and are globally interconnected. Research institutes—like those of France’s *Centre national de la recherche scientifique* (CNRS) or Germany’s Max Planck Society—though founded in the 20th century, are similarly well-established. The countries differ in the time elapsed since establishment and in the differentiation of these organizational forms and fields. Comparing the four research university sectors, Germany and Belgium are more highly institutionalized than France and Luxembourg. By contrast, in research institutes, France and Germany have large, differentiated non-university research sectors.

We begin with process-tracing in each country, based on synthesis of the research literature in multiple languages and emphasizing the founding dates of organizations and system institutionalization. Process-tracing helps us to understand sequential (historical) events and allows us to explore developmental processes in specific cases (Mahoney 2004: 88f). We pair the historical case analysis with quantitative analysis of bibliometric data. This combination facilitates analysis of how these organizational forms and fields evolved and provides results on their scientific productivity.

### Germany’s Research Universities and Extra-University Research Institutes: Two Pillars of Strength in Science

Germany is home to the undisputed model of the research university and significant extra-university research institutes. Yet universities have been underfunded for decades (Pritchard 2006), despite considerable increases in the proportion of each cohort entering higher education, and the sector is divided into two, with research universities and universities of applied sciences. Paradoxically, policymakers have ignored this “institutional crisis” of underfunding higher education even as they send ever more of their children into the system (Lenhardt 2005): “Stagnation of public funds is particularly damaging to efforts towards fostering internationally competitive basic research in the universities, as they receive only a relatively small share of the entire national research budget” (Baker 2014: 93). Here, there is a decoupling between policy rhetoric and reality.

Indeed, the German “Humboldtian” model of university-based science is among the oldest and influential conceptions of higher education worldwide (Humboldt 1809), reaching mythic proportions, despite the on-going transformation of German higher education—not least the reunification that led to unforeseen, dramatic dynamics in academia (see Ash 1999; Clark 2006; Pritchard 2006). While the foundational principle of the nexus of research and teaching enjoys sustained attention worldwide, the relationship remains complex and ambiguous both within organizations and between the organizational fields of higher education and research. The success story of German research-based teaching relies on self-government, institutional and organizational growth, and its generality, dealing with matters of general human interest and preparing students for a broad range of occupations (Ben-David [1977] 1992).

Germany’s 126 research universities, 232 universities of applied sciences, and 51 art and music colleges operate alongside a research-intensive and powerful extra-university research institute sector of around 300 institutes, most gathered in four large umbrella associations. With annual R&D investments among the highest in Europe (OECD 2016), the Federal Ministry of Education and Research (BMBF) is the key actor in research policy, even as education is mostly the province of the *Länder*. Among public funding organizations, the German Research Foundation (DFG) is the main promoter of science. Furthermore, the European Commission and more than 16,000 foundations offer innumerable possibilities to apply for financial support for education and research (Hinze 2010).

Higher education devoted to research grew stronger in Germany than in more differentiated systems like that of France. This research-focused type of university continues to dominate German higher education up to today, despite establishment of universities of applied sciences (*Fachhochschulen*) after massification of tertiary education. Since the 1960s, this new organizational form provides a more applied and praxis-oriented focus. Investments in (fundamental) research are less significant; however, increasingly their faculty members do conduct research, often collaborating with industry. They have become more like research universities, even if the monopoly on granting doctoral degrees remains in universities (Teichler 2005).

Around WWI, Germany established an alliance between representatives of science, research-intensive industry, and ministerial bureaucrats to found innovative research institutes outside universities. The 1911 founding of the *Kaiser-Wilhelm-Gesellschaft* (from 1948 Max Planck Society) challenged the German higher education system as the dominant locale for fundamental research. In this sector, research was institutionally separated from teaching. Today, 83 Max Planck institutes are located in Germany and 5 institutes abroad; the newest—the Max Planck Institute for International, European, and Regulatory Procedural Law—opened in Luxembourg in 2012. After WWII, further competitors entered the growing organizational field of extra-university research: The Fraunhofer Society was established in 1949 to focus on applied sciences and industrial contract research (today: 67 institutes). The Leibniz Association was established in 1997, but had existed since 1977 known as the “blue list” (*Blaue Liste*), a collection of diverse research institutes with regional or national significance and varying emphases on fundamental or applied research (today: 89 institutes). The Helmholtz Association of German Research Centers (2001), dealing with research related to infrastructure (*Vorsorgeforschung*) comprises 18 very large research institutions (*Großforschungseinrichtungen*) and around 40 federal research institutions in a range of fields related to national interests (Hohn 2010). Yet all of these research institutes and their umbrella associations continue to rely on universities for crucial aspects of their work, whether it be training of young scholars or certifying doctoral candidates. Thus, the competition must be considered more of a symbiosis, with elements of collaboration and competition continuously (re)negotiated.

**Fig. 3:**
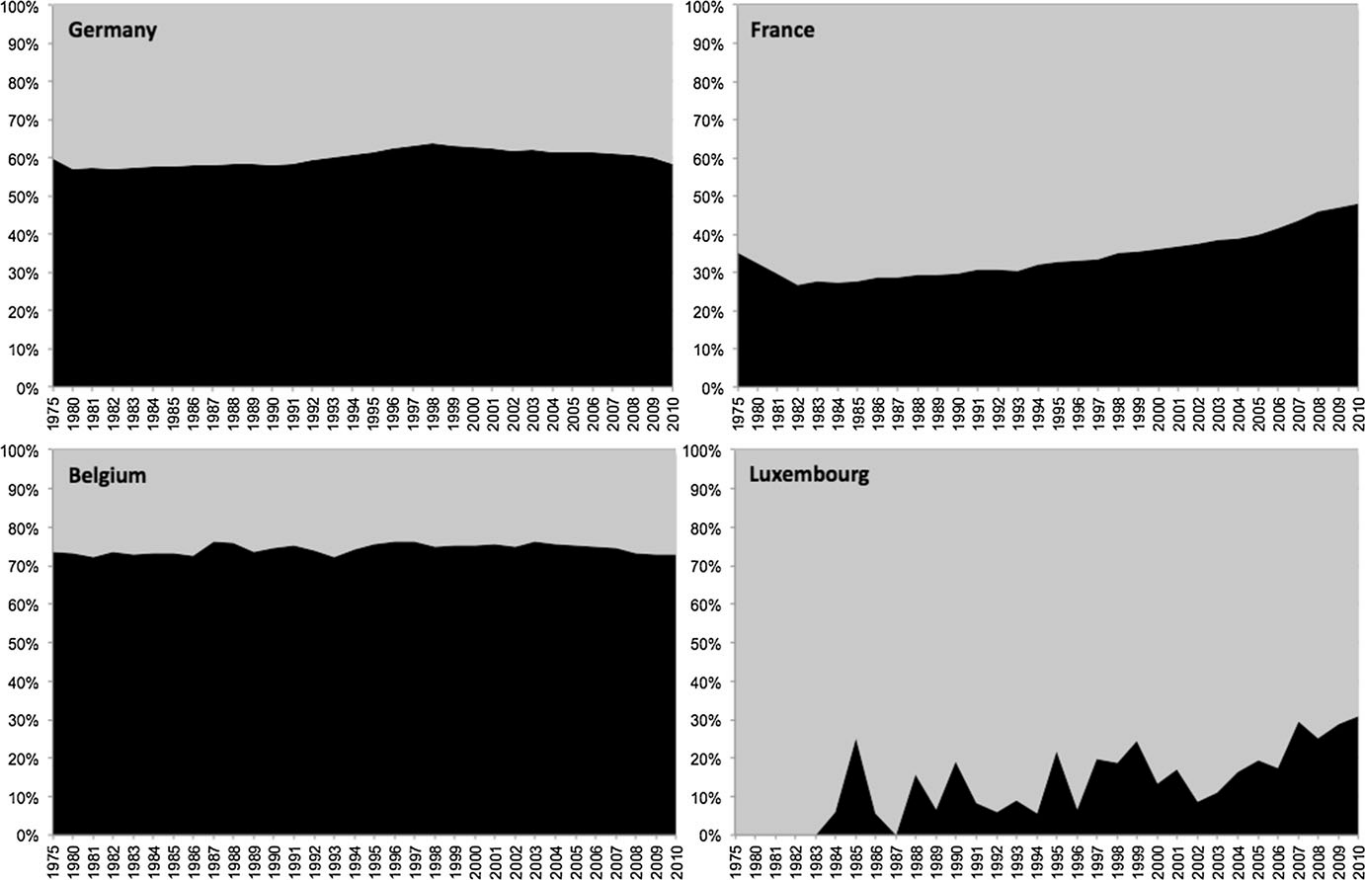
Scientific Productivity in Germany, France, Belgium, and Luxembourg: Universities vs. Non-university, 1975–2010. Note: This representation is based on differentiating publications from organizations with the word “univ” from all others, thus is a rough measure of the productivity of the university vs. non-university sector, which includes a range of science-producing organizations, including firms, large government agencies (e.g., in France), and academies (e.g., in the GDR). “*Black*” = university sector, “*grey*” = non-university sector. *Source* Authors’ database of SCIE publications based on Thomson Reuters’ Web of Science

Universities have come under pressure due to declining funds and internationalization and Europeanization processes. Competition between universities and research institutes has increased as centers of excellent research outside universities intensify their activities, increase investment in cutting-edge research projects, and amass the best and brightest scientists. Their enviable funding derives from both Federal and *Länder* governments jointly providing funding, though in differing proportion (usually 50/50) (Hohn 2010). Germany’s dual pillars of mass universities and independent research institutes continue to boast prodigious scientific output—and the universities’ central position has been maintained (see Fig. 3).

With the emergence of newer path-breaking hybrid types of research (and teaching) as well as universities of applied science demanding the right to confer doctoral degrees, the German higher education system confronts a new situation. The structural duality of the German system no longer seems unassailable or as sustainable. Examples of newer boundary-spanning organizations include the Karlsruhe Institute of Technology (KIT), an amalgam of the Karlsruhe Research Center (Helmholtz) and the Technical University of Karlsruhe, as well as the International Max Planck Research Schools (IMPRS) as examples for inter-institutional, international, and interdisciplinary collaboration (www.mpg.de/de/imprs). Because only research universities in Germany have granted doctoral degrees, others depend on close collaboration or “strategic partnerships.” Furthermore, this concept has been affected by three developments: massification, segregation of research and teaching, and growing third-party funded research.

Having demonstrated the continued strength of Germany’s two main pillars of research capacity, we turn now to France, also exhibiting structural duality.

### France: Elite Professional Higher Education and Academic Drift in Universities

France’s differentiated higher education system consists of a range of universities, some very strong in research and others more focused on teaching and applied fields. Universities are challenged by the elite higher professional schools, the *grandes écoles,* to attract talent. And in research the *Centre national de la recherche scientifique* (CNRS) is dominant, though many of its researchers establish or work in research laboratories (research groups) physically located within universities. France finances and maintains prestigious extra-university research units and institutes, many, but not all, under the CNRS umbrella. With 79 universities, 205 *grandes écoles*,^2^ and 14 foreign institutions, the professional school sector remains significant (METRIS 2012c). The Paris-Sorbonne University was among the first universities in Europe; for centuries the guarantor of academic excellence across diverse fields. Today’s major concentration of universities in the capital city is built upon those ancient foundations. In 1970, shortly after the student protests of 1968, this institution was decentralized and divided into 13 autonomous universities (Musselin 2007: 713). The national extra-university sector consists of seven larger umbrella research associations with more than 70 institutes, centers or departments. Most recently, in what Musselin (2017) calls the remodeling of French higher education, consortia are being created that are to grow collaborations across organizational forms and aggregate research in stronger groupings of researchers and organizations. At Paris-Saclay, for example, bridges are being built between 18 research organizations, including two universities, an *Ecole Normale Supérieure*, six research organizations, ten engineering and business schools, and two educational clusters that host 10,000 researchers and 300 laboratories.^3^ Clearly, the underlying theory is that physical proximity matters for scientific exchange; however, this does run counter to some important features of the recent decades, namely, globalization and intellectual exchange via virtual communication platforms.

Despite the principle of equivalence, France’s tertiary education and research system exhibits stratification: the *grandes écoles*/university divide and the split between selective and non-selective segments as well as distinctions between CNRS researchers and academy members at the top and regular university faculty members below. While the key organizational form for research may be—increasingly—the university, CNRS laboratories and institutes play a key role within them and more generally in producing science. The *grandes écoles* constitute a diverse group of highly selective and prestigious institutions that train future elites: higher-level civil servants, professors and researchers, engineers, and company managers (Givord and Goux 2007), but increasingly they also produce science (OECD 2014). Widely criticized, this divide has often been blamed for the current crisis experienced by universities, as the *grandes écoles* attract high-achieving students and relegate universities to struggle for global reputation (Clark 1995: 93). Thus, from 2006 ‘alliances’ have been formed to join both organizational forms, such as in Centers for Research and Higher Education (PRES) (Le Deaut 2013).

The contemporary university crisis also results from lack of resources, multiple incoherent reforms, lack of labor market forecasting, and increased bureaucratization (Bernhard 2017). Universities’ status is limited because neither are societal elites trained there nor are the most significant research projects initiated by them, thus they serve mainly as teaching bodies, even if some host influential research groups or laboratories. With notable exceptions and shifting recently, both *grandes écoles* and universities emphasize teaching more than they excel at research. The French higher education system reflects an “education model,” emphasizing professional preparation (Kreckel 2008: 88).

Yet French universities are changing, not least due to global norms and European standards. Universities were responsible for general education (except for law, medicine, and pharmacy), while *grandes écoles* offered vocational preparation of elites or middle-range technicians. Research was long conducted primarily in separate research organizations. A fundamental shift, the Liberties and Responsibilities of Universities (LRU) bill, passed in August 2007, grants significant power to university presidents. The proclaimed aim: to meet the demands of the “knowledge economy” and to bring French universities to the level of excellence of major international competitors. The French “excellence initiative,” designed to strengthen research collaborations and consortia of researchers within a differentiated higher education system, cannot eliminate decades of specialization and uneven development. Ironically, the German Excellence Initiative aimed to do the opposite, creating more differentiation in a less stratified, less differentiated higher education system (Münch 2007). Along with the diffusion of “performance discourse” and new instruments such as “agencification” came national calls designed to identify the best researchers and encourage their collaboration; yet perhaps most significant is the requirement that all universities must be part of scientific consortia (Musselin 2017).

Historically, some processes have successfully linked teaching and research in France. In the late 19th century, the new organizational form of *grands établissements* was established to support and develop training and research, including the *École pratique des hautes études* (1868) and the *Institut Pasteur* (1887), which has grown in capacity and influence (Hage and Mote 2008). Founded in 1530, the *Collège de France* enjoys special status among the *grands établissements* (Kreckel 2008).

Since 1939, fundamental research is predominantly financed by CNRS, the dominant association of research institutes, units, and laboratories. This state-funded, complex umbrella organization encompasses seven research institutes, three national institutes and 1,028 research units, with the vast majority (95%) joint laboratories with universities and industry. CNRS is significant for France’s scientific development and international standing in a wide range of fields. Organized in associations, university faculty members may apply to establish collaborations with one of the national research institutes, or associated laboratories receive funding, and sometimes CNRS staff, while autonomous research units—called *unités propres—*have no university affiliation (Musselin and Vilkas 1994: 129). The varying relationships of researchers to each other and the organizational forms in which they work confounds analyses of affiliations and aggregate measurement of the impact of organizational conditions on outputs for France.

Other publically-funded extra-university research institutions conduct strategic research related to national needs, from infrastructure and energy to agriculture and health, all part of a powerful centralized state (Clark 1995). The funding and organization of research has traditionally been the responsibility of separate organizations; with the institutional separation between higher education and research difficult to bridge (Ben-David [1977] 1992: 107)—and continuously debated. Yet, this is precisely what French research policy seeks to accomplish today in establishing consortia connecting organizations and research groups. The traditional government-sponsored, largely autonomous research organizations operate alongside competitive project-based funding in large competitive programs (OECD 2014). Thus, higher education and research and development remain particularly complex in France, despite efforts underway to enhance coordination and consolidation (Musselin 2017).

Turning now to output, we examine France’s overall scientific productivity in STEM, and find continuously rising output and strengthened university-based research. The non-university/university sectoral divide has been narrowed (see Fig. 3). These sectors’ output grew in parallel for decades, witnessing considerable expansion from 2004 onwards. Today, the two pillars of French science are at parity, at least in terms of STEM article publications. Importantly, institutional affiliations in France tend to be multiple, with many CNRS researchers working within universities and universities collaborating with national research institutes. Furthermore, universities have different branches for different fields, but the actual organizational setting in which the research was produced is not always distinguished. In bibliometric databases, such as Thomson Reuters’ Web of Science, the primary affiliation is paramount. A methodological challenge that qualitative research should address at the organizational level is how are resources provided within these settings and what reputational logics guide noted affiliation.

Thus, in both France and Germany, extra-university research institutes play an important role in research, as do the associations that directly fund research by selecting the best scientists and providing them with research-conducive conditions. Despite the dual structure that places emphasis and concentrates resources in the research institutes, with varying degrees of independence and collaborations across the institutional divide, both enjoy significant capacity and output.

Turning now from two very strong mid-sized science-producing countries to two smaller countries, Belgium and Luxembourg have undergone significant transformations in higher education and research through European and within-nation policy interventions. Belgium exhibits considerable endogenous dynamics given the internal cleavages that exist, from language and religion to geography. Despite hosting the key European capital city, Belgium faces political challenges in maintaining a functioning nation-state. Next to Belgium, Luxembourg shows considerable diversity in languages as does its Flemish- and French-speaking neighbor. Over centuries, stemming from its modest size, the Grand Duchy has been majorly influenced and affected by the countries with which it shares roots and borders. Socially and demographically, the small state Luxembourg is hyper-diverse and growing rapidly, reflected in science as well (Meyer 2008). These contextual factors crucially affected the institutionalization of higher education and research that provides conditions and capacity for science today.

### Belgium’s Strong Research Universities Reflecting Social and Political Cleavages

Due to its cultural and political history, the Belgian education landscape is divided into language communities also responsible for higher education and research policies. The two largest communities are Flemish-speaking Flanders and French-speaking Wallonia. A small group of German-speaking Belgians live mainly in Eupen. Brussels, the cosmopolitan European capital, provides a central meeting place for these communities (Dassen and Luijten-Lub 2007: 9f).

Belgium has six state-funded older major universities (University of Liège and Ghent University), Catholic (Catholic University Leuven and Catholic University Louvain) or free (*Université Libre de Bruxelles* and *Vrije Universiteit Brussel*). In 2003, two public institutions, the University Institute Antwerp (UIA) and the State University Centre Antwerp (RUCA) and the private University Faculty Saint Ignatius Antwerp (UFSIA) were merged to form the University of Antwerp. Students in Flanders can also study at the Dutch Open University in the Netherlands or the Transnational University of Limburg, a cross-border university merger of the University of Hasselt and the Netherlands’ University of Maastricht (van Petegem and Imbrecht 2012: 132). A European education hub, Belgium maintains six Flemish-speaking and seven French-speaking universities (METRIS 2012a: 31).

Professional and technical higher education in Belgium is not university based; instead, this is provided by 22 *hogescholen* in Flanders and 21 *haute écoles* in Wallonia (METRIS 2012a). In 2003, structural reforms in Flemish higher education created “associations” and a binary system with professional Bachelor degrees offered by university colleges and academic Bachelor and Master degrees offered by universities (Huisman and Mampaey 2016). In both Flanders and Wallonia, universities maintain the monopoly on granting doctoral degrees. In Wallonia, universities are associated in academies: the Louvain Academy, the Wallonia-Brussels Academy, and the Wallonia-Europe Academy (BMC 2009: 46).

Since 1874, fundamental scientific research has been an exclusive part of universities in Belgium.^4^ Applied scientific research has also been conducted by researchers in *hogescholen*, preferably in tight collaboration with universities. Financing is provided by industry, government, and through general university funds and other research funding. Although many research institutes are linked to universities, Flanders provides independent extra-university research institutes funded by the government and business enterprises (Dassen and Luijten-Lub 2007: 33ff). Four major strategic research centers in Flanders are: Interuniversity Microelectronics Centre (IMEC, since 1984), Flanders Institute for Biotechnology (VIB, since 1995), Interdisciplinary iMinds (since 2004), and Flemish Institute for Technological Research (VITO, since late 2000s) (BMC 2009: 34; see also Belspo 2013). In Wallonia, from 2002, a network of seven science and technology parks host high-tech companies and support relationships between their tenants and university researchers. Currently, they comprise more than 500 companies and employ 13,000 people (www.spow.be).

Regardless of the different structures in parts of Belgium, the majority of Belgium’s scientific output results from centuries-old and newer strong research universities.

### Luxembourg: Building Capacity through Public Research Centers and a New Research University

Situated in the heart of Western Europe, Luxembourg long relied on other countries to provide most higher education and advanced research. While Luxembourg’s capacity in higher education and research has risen rapidly, it remains limited in comparison to the other countries. Luxembourg, like Belgium and France, hosts one of the three European Union capital cities, and thus symbolizes European and global goals. Decision-makers rhetorically support investments in higher education and science, yet national policymakers have recently limited the planned growth of R&D, favoring consolidation. The Bologna process considerably intensified the on-going standardization in European higher education, with Luxembourg signing the declaration years before the University of Luxembourg (UL) was founded (2003)—built upon several organizations that provided teacher training, conducted research, and served national priorities. Scientifically, strategic investments in promising research areas of national priority are meant to compensate being a new, small university (Meyer 2008; Powell 2012). Transforming research policy, this establishment marked a significant break with the past; UL ranked 193 in the THE World University Rankings 2015–2016.

Luxembourg’s small, but diverse higher education system is matched by a number of research institutes and medical facilities active in various scientific fields. Consolidation in the publically-funded research sector aims to achieve (even) better results, although given the investment timeframe and the quick rise in productivity, it is too early to conclusively measure the effects of capacity-building efforts. In January 2015, two Public Research Centres—Henri Tudor and Gabriel Lippmann—merged to form the Luxembourg Institute of Science and Technology (LIST), designed to achieve critical mass internally and strong visibility externally. Several years ago, a new contract stipulated that the UL should collaborate with the country’s public research institutes in research areas thought to develop and diversify the economy. The 3LIU consortium includes the University, LIST, Luxembourg Institute of Health (LIH)—a hybrid of the CRP Health and the Integrated Biobank of Luxembourg—and the Luxembourg Institute of Socio-Economic Research (LISER). The research university is the centerpiece of the science system, with strong ties to all the other research institutes and funding bodies, now mainly sharing Campus Belval.

The organizational field of research in Luxembourg has grown substantially over time (Meyer 2008; LuxInnovation G.I.E. 2009; METRIS 2012b; OECD 2016). Yet the absolute overall production in SCIE publications remains limited in comparison. Beginning in the 1980s, an innovation and research support policy was formulated, with the establishment of the National Agency for Innovation and Research. Since 1987, laws have established public research centers, explicitly included incentives for R&D, and supported private-sector research activities. Founded in 1999, the Ministry for Higher Education and Research defines, coordinates, and implements R&D and innovation policies.

Although Luxembourg’s policymakers attempt to diversify the economy beyond financial services and to improve R&D infrastructure and bolster conditions for innovation, some question whether the country has sufficiently endowed its university and strengthened its research institutes given European and global competition in tertiary education and science (Meyer 2008; OECD 2016). The Grand Duchy continues to strengthen its scientific networks with neighboring countries (e.g., in the *Université de la Grande Region*) and globally, a key advantage.

While the national university is the leading single organization in scientific output in natural sciences, the research institutes and hospitals together produced more articles. Given this duality in terms of research output, Luxembourg’s state-funded science output derives from both pillars, as in Germany and France. We now turn to explicitly compare scientific productivity in the four countries.

## Rising Scientific Productivity across Western Europe and the Research University

The over-time and cross-national comparisons emphasize that Germany, France, Belgium, and Luxembourg, as larger and smaller neighboring countries embedded in the EU, have contrasting policies in R&D and varying investments and proportions of scientists of all employees. Their higher education and research systems reflect different institutionalization pathways and combinations of universities and institutes, each organizational form contributing more or less to scientific productivity. In each country, research universities and research institutes (often gathered in umbrella associations) contribute different proportions to overall scientific output, but in all four countries the research universities represent the key organizational form.

**Fig. 4:**
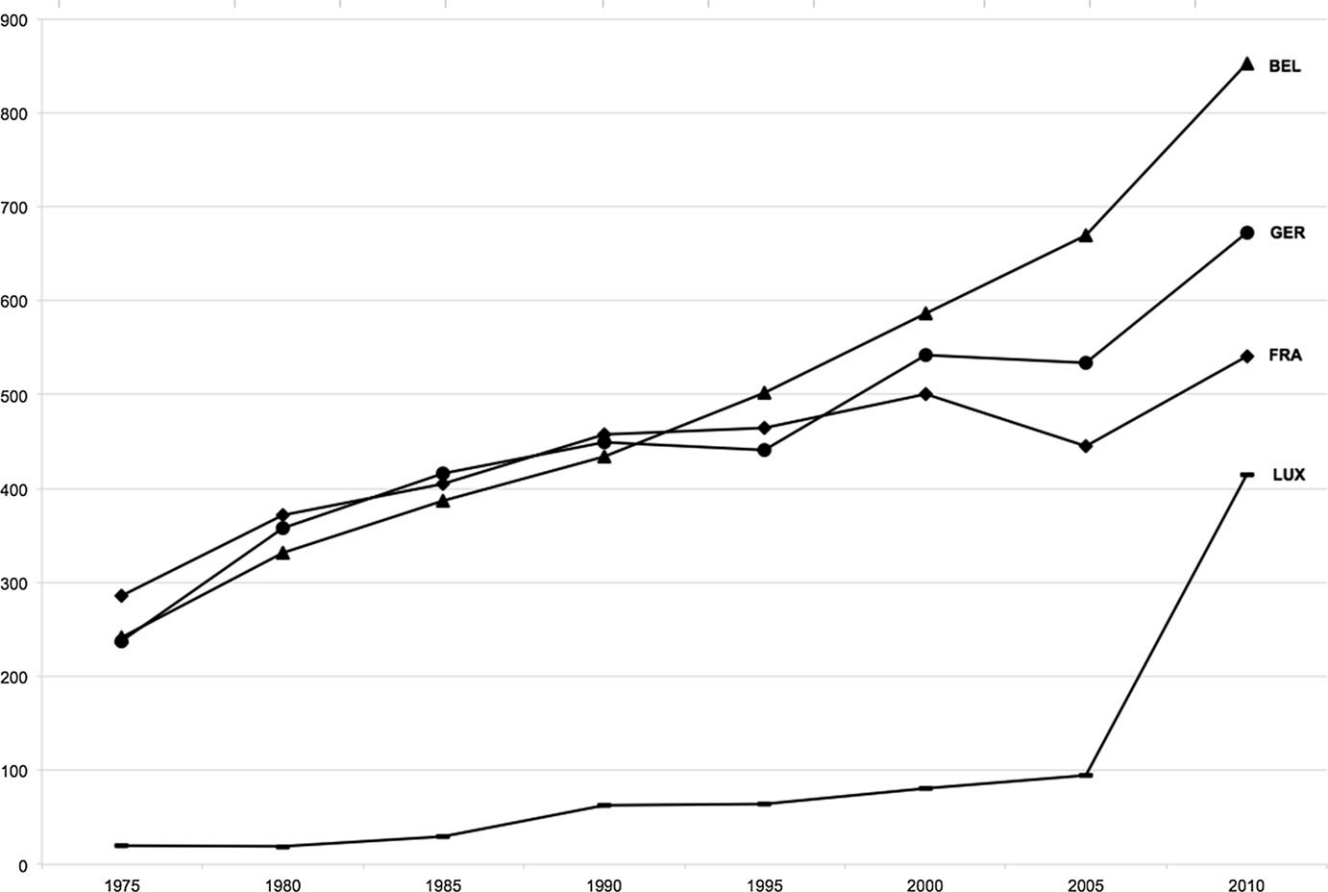
Number of Publications from Germany, France, Belgium, and Luxembourg (per million inhabitants), 1975−2010. *Sources* Authors’ database of SCIE publications based on Thomson Reuters’ Web of Science; OECD.Stat (2017): Main Science and Technology Indicators; last accessed 2017.06.08

The total number of SCIE publications for the four countries over the 20th century shows massive increases, especially since the 1970s and again over the past decade. With major differences in scale, Germany and France have increased their output dramatically over the past four decades. Belgium’s production has grown strongly as well, but more evenly. Luxembourg, given its small size and later investments in R&D, must identify particularly promising areas if it hopes to enhance its more qualitative than quantitative contribution to global science. As absolute numbers are difficult to interpret across cases of different size and science capacity, we calculated the scientific output per one million inhabitants (see Fig. 4). This enables us to more reliably measure the productivity based on SCIE publications in leading journals. While the long-term scientific strength of Germany (even during the division of West and East Germany) continues to the present day, it is Belgium, with its group of powerful research universities, that leads in per capita productivity, followed by Germany, France, and Luxembourg (all relatively similar, with Luxembourg catching up through university expansion).

Comparing the absolute production levels of countries historically manifests the dramatic rise of science. The four countries examined here have, since the 1980s, witnessed a veritable boom in the publication of scientific articles in STEM disciplines as well as health. Comparing cases of very different size, we must, of course, acknowledge issues of scale and scope. When analyzing the number of these publications per 1,000,000 inhabitants, we find an even more dramatic rise, especially over the past decade. This productivity measure provides more comparable indicators and also reorders the countries. In contrast to the investments, viewed in “research intensity” (Fig. 1), in terms of SCIE publications per million inhabitants, Belgium has by far the highest productivity, followed by Germany, France, and Luxembourg. Resources alone can fully explain neither the expansion nor the country-level differences found. Indeed, Luxembourg, spending less than half as much as its neighbors, has built capacity effectively in strategic fields. With targeted investments, Germany after reunification continued its growth pattern (the GDR had stable but low productivity; see Fig. 4).

Our study investigated the contributions of different research organizational forms to scientific productivity. We compared the production of STEM research in four larger and smaller countries in Europe. As presented earlier (Fig. 2), these countries achieve their scientific outputs having distinct and differently institutionalized higher education and science systems. Germany has long-established research universities and institutes that produce a large number of articles—more than do the equivalent organizations in aggregate in France, Belgium, and Luxembourg. France, while relying on a group of strong universities, emphasizes teaching and has fewer such organizations than does Germany. But France also funds a diversity of well-established research institutes and other organizational forms, including the influential and highly-productive CNRS. Still, France follows Germany in the total number of publications. Belgium has few research institutes; its capacity mainly relies on several important universities. Taking Flanders, Brussels, and Wallonia together, Belgium is the leading country of these four, if we measure publication productivity relative to population.

Our key finding is that the institutionalization of the research university sector and reliance on it supports high productivity (see Fig. 2). In fact, those large and dual structured systems with a highly institutionalized non-university sector, as in France and Germany, have less per capita output than Belgium, with its highly-developed and well-funded university sector. Luxembourg, with its recently-founded research university and several research institutes, while catching up quickly, cannot yet match the other countries. This also strengthens the small state thesis—of adaptability and comparative advantage—found in other parts of Europe (Meyer 2008). Smaller European countries in which basic research is mainly done in universities are relatively more productive than the mid-sized or even the largest science producers that have strong non-university sectors receiving considerable resource shares (May 1997). Small states rely heavily on a few strong research universities institutionalized over decades if not centuries—and must capitalize on their strengths. Cole and Phelan (1999) have argued that wealth strongly but not completely influences the volume of research produced by countries. Indeed, the proportion of researchers in these countries varies marginally, from 9.7 per 1,000 employees in Belgium to 9.2 in France, 8.6 in Luxembourg and 8.4 in Germany (OECD 2016). Differences between these four wealthy European countries in scientific productivity cannot be fully explained by differences of overall investments in science. Rather, the institutionalization and distribution of organizational forms in which researchers are producing science remain crucial factors to be examined further.

Examining four contributors to the European center of science productivity, we found remarkable sustained growth, building on the evolving institutionalization of research universities and institutes and embeddedness in worldwide scientific networks. The elaboration of scientific communication through a world of scientific journals built upon peer-review and rising (inter)national competition and collaboration in STEM fields spur global growth—with Europe still central to global science.

A surprising result is that the most established and high-volume absolute producers, Germany and France, have been outpaced by Belgium, with small state Luxembourg catching up in scientific productivity per capita (on a much lower level of absolute production). More investment in R&D does not necessarily yield more STEM research in international, English language journals indexed by Thomson Reuters’ Web of Science database, although its coverage is steadily growing. Technological change that facilitates communication and collaboration, internationalization, and the global *lingua franca* of English in which research must be reported in leading SCIE journals affect scientific output as well as the attention paid to particular research or the measurement of productivity. More generally, the conditions of the research enterprise and the publication strategies of individual researchers have shifted, with these factors requiring further fine-grained analysis.

In investigating what makes these European countries successful in science, we identified the long-term development yet differential elaboration of research universities and research institutes. Turning to these diverse organizational forms, we unsurprisingly find dual pillars of strength in science—research institutes and research universities—in France and Germany, but the dominance of research universities in all four countries. Regarding the unique contribution of universities, this organizational form, despite its relatively modest proportion of funding, remains the key contributor of STEM publications. In contrast to the resource-dependency argument that systems with strong research institutes without teaching obligations and funds primarily devoted to research should be more productive, we show that it is those systems relying on strong university sectors that are relatively more productive.

In cross-national and historical comparison, neither solely size of country nor level of R&D investments account completely for the growth of scientific productivity. While newer entrants to the world of science can quickly increase their capacity and productivity by investing heavily in research infrastructure and recruiting talent worldwide, especially the older established universities and associations of research institutes have successfully driven the considerable increase in science production over the last several decades.

The exploratory historical and comparative research reported here uncovered not only huge growth historically, but also relatively stable patterns of productivity of the universities within countries, albeit with growth in France and even more in Luxembourg. Next steps in understanding better the publication patterns—not only in the fields of science, technology, engineering, mathematics, and health—include analysis of the size and international networks of communities of researchers, the contributions of various organizational forms in the diverse non-university sector, including firms, and organization-level studies of those most productive organizational forms—most centrally the research university.

## Footnotes

1 We cannot here address in-depth ancillary questions of disciplinary differentiation, industrial investment in R&D or the fluctuating influence of academies of science.

2 Disagreement persists about the number of *grandes écoles*. The *Conférence des Grandes Écoles* notes 205 *grandes écoles* in France (http://www.cge.asso.fr/en/our-members/grandes-ecoles, last accessed 2016.08.10).

3
https://www.universite-paris-saclay.fr/en.

4
http://education.stateuniversity.com/pages/155/Belgium-HIGHER-EDUCATION.html, last accessed: 2015.08.11.
